# The Value of Neutrophil Cell Population Data Parameters as Markers of Systemic Inflammation in Dogs and Cats

**DOI:** 10.1111/vcp.70029

**Published:** 2025-06-06

**Authors:** Dylan S. O′ Toole, Tim L. Williams, Cassia H. Z. Hare

**Affiliations:** ^1^ Department of Veterinary Medicine University of Cambridge Cambridge UK

**Keywords:** acute phase proteins, biomarkers, CPD parameters, neutrophils, systemic inflammation, toxic change

## Abstract

**Background:**

Neutrophil cell population data (CPD), including fluorescent light intensity (NE‐SFL) and side scatter (NE‐SSC), are promising inflammatory markers in human sepsis but remain unexplored in dogs and cats.

**Objectives:**

Determine the diagnostic utility of NE‐SSC and NE‐SFL for detecting systemic inflammation in dogs and cats.

**Methods:**

Dogs and cats with archived CPD, blood films, and acute phase protein (APP) measurements were included. Increased C‐reactive protein (CRP) in dogs and Serum Amyloid A (SAA) in cats were considered indicative of systemic inflammation. CPD was compared with APPs, white cell count (WCC), neutrophil count, band neutrophil count, and toxic change grade. Optimal cut‐offs and associated sensitivities and specificities were calculated using ROC curve analysis. Correlations were assessed using Spearman's coefficient.

**Results:**

NE‐SFL and NE‐SSC were significantly increased in dogs and cats with systemic inflammation. The area under the curve (AUC) of NE‐SFL was higher than that of NE‐SSC, WCC, and band neutrophil count in both dogs (0.82) and cats (0.77). The optimal NE‐SFL cut‐off for detecting systemic inflammation was > 41.7 ch in dogs (sensitivity 80%; specificity 66%) and > 37.4 ch in cats (sensitivity 75%; specificity 67%). NE‐SFL was positively correlated with APPs, WCC, neutrophil count, and band neutrophil count in both species. NE‐SSC was positively correlated with APPs in both species and, in dogs, also with WCC, neutrophil count, and band neutrophil count.

**Conclusion:**

CPD, particularly NE‐SFL, is a promising marker of inflammation in dogs and cats and could be especially useful when APP quantification or blood smear examination are unavailable.

## 1 Introduction

In humans, markers of systemic inflammation include leukogram changes such as alterations in neutrophil number and morphology, positive acute phase proteins (APPs) such as C‐reactive protein (CRP) and serum amyloid A (SAA), and cytokines such as Interleukin‐1 beta (IL‐1 beta), Interleukin‐6 (IL‐6), and Tumor Necrosis Factor‐alpha (TNF‐alpha) [1]. Studies in humans have shown that morphological changes such as left shift and toxic change in neutrophils have lower combined sensitivity and specificity for detecting systemic inflammation and infectious disease than APPs such as CRP [2], while cytokines (which are not readily available in veterinary medicine) have been shown to have comparable sensitivity and specificity to APPs for detecting systemic inflammation [3, 4, 5]. In dogs and cats, systemic inflammation can similarly be detected by quantifying shifts in leukocyte number, the presence and severity of left shift and toxic change in neutrophils on blood smear analysis, and detection of elevated serum APP concentrations [6, 7, 8, 9], with the latter again regarded as the superior markers of systemic inflammation [10]. Indeed, even among board‐certified clinical pathologists, there can be significant interobserver variability in the identification of band neutrophils and grading of neutrophil toxic change [11]. Furthermore, APPs are expensive to measure, which limits their widespread use [12, 13]. Similarly, cytokines are rarely used as markers of systemic inflammation in dogs and cats, likely due to their instability and the challenges associated with measuring them in the clinic [1]. As such, there is continued reliance on readily available hematological parameters, which, as in humans, are limited in their diagnostic sensitivity and specificity. For example, neutrophilia and lymphopenia can be induced by glucocorticoid therapy and stress, independent of systemic inflammatory disease and infection [14, 15]. Additionally, there is inherent subjectivity and intraobserver variability in detecting and grading morphological changes in leukocytes, such as toxic change in neutrophils, and these are also lacking in mild inflammatory disorders [11].

Laser‐based hematology analyzers such as the Sysmex XN provide a 5‐part differential count and scattergrams, which can detect abnormalities in the differential count [16]. Other potential markers of inflammation in humans, which have generated significant interest, are neutrophil cell population data (CPD) parameters [17]. These parameters are measured via the white blood cell differential fluorescence (WDF) channel of the Sysmex XN hematology analyzer, which discriminates individual cells using a combination of fluorescence and flow cytometry [18]. Pores are created in leukocytes using surfactant and a fluorescent dye, then stain nucleic acids and cytoplasmic organelles [16]. Two such CPD parameters are neutrophil side scatter (NE‐SSC), which is influenced by intracellular complexity and granularity, and fluorescent light intensity (NE‐SFL), which is influenced by cellular protein/nucleic acid content [18]. CPD is recorded, numerical values that provide information regarding both the functional and morphological characteristics of neutrophils [18]. An increase in NE‐SFL is hypothesized to represent an increased proportion of circulating, immature neutrophils, which have higher RNA/DNA content and therefore higher fluorescent intensity [17]. In contrast, there is limited evidence to suggest that NE‐SSC is associated with neutrophil toxic change, and this warrants further investigation [19]. While these changes are apparent upon visual appraisal of the WDF scattergram, based on the authors' experience, the interpretation of scattergrams is subject to user variability, and therefore, objective measures of scattergram data such as NE‐SFL and NE‐SSC aid in interpretation.

Studies that have explored the usefulness of neutrophil CPD parameters in humans have evaluated their ability to detect sepsis [20, 21]. Notably, however, these CPD parameters are not yet implemented routinely in the clinic. NE‐SFL has been found to have good diagnostic sensitivity for detecting sepsis, and higher NE‐SFL values are associated with poorer clinical outcomes [21]. Another study of human patients with bacterial infection showed that NE‐SFL had moderate diagnostic performance for indicating bacteremia, and a weak positive correlation with bacterial loads based on PCR was observed [17]. To date, the inflammatory biomarker potential of NE‐SSC in veterinary patients with sepsis has yet to be explored. CPD parameters are attractive inflammatory biomarker candidates, which may indicate the need for additional laboratory testing. Furthermore, results are rapidly generated at no additional cost to the patient.

Given the value of CPD parameters in detecting sepsis in humans, we hypothesized that neutrophil CPD parameters would also be promising inflammatory markers in dogs and cats. The aims of the current study were (1) to determine the diagnostic sensitivity and specificity of NE‐SSC and NE‐SFL for detecting systemic inflammation; (2) to compare the diagnostic sensitivity and specificity of NE‐SSC and NE‐SFL to established markers of inflammation; (3) to determine the intra‐assay variability, interassay variability, and effects of sample aging on NE‐SFL and NE‐SSC.

## 2 Materials and Methods

### 2.1 Case Selection and Criteria

This retrospective observational study included dogs and cats that presented to a veterinary referral hospital between June 2022 and June 2023 and was approved by the local Research Ethics Committee (CR723). The inclusion criteria were (i) dogs and cats that had a CBC performed on the Sysmex XN‐V hematology analyzer between June 2022 and June 2023; (ii) an archived blood smear available for review; and (iii) APP measurements (CRP for dogs and SAA for cats, according to laboratory profiles offered at that time) recorded on the same day as the CBC and blood film. All cases matching these criteria were included. Cases were excluded if the data were lacking or performed across different days. In cases where repeated clinical pathology data were available for the same patient at different time points, only the sample collected on initial presentation was included. Data from dogs and cats were analyzed separately.

### 2.2 Data Collection

Patients matching the inclusion criteria were identified via a search of the Laboratory Information Management Systems (LIMS). Automated white blood cell count (WCC), automated neutrophil count, and serum SAA and CRP concentration were recorded from LIMS data.

### 2.3 Hematology and Biochemistry Data

Hematology values were previously obtained using a Sysmex XN‐1000 V analyzer (Sysmex Corporation, Kobe, Japan), and biochemistry results were obtained using a Beckman AU480 analyzer (Beckman Coulter, Brea, USA) [22, 23]. The CPD parameters, neutrophil side scatter (NE‐SCC) and neutrophil fluorescent intensity (NE‐SFL) were recorded for each patient from the WDF tab of the Sysmex XN‐V service menu. The observer was blinded to CPD parameters, other analyzer data, and the SAA or CRP concentration during data collection.

### 2.4 Confirmation of Systemic Inflammation in Dogs and Cats

Serum CRP (for dogs) and SAA (for cats) concentrations were determined using previously validated automated human immunoturbidimetric assays (Randox CRP and Eiken SAA) [22, 23]. Based on these previously validated assays, a serum CRP cut‐off of > 6.8 mg/L and a serum SAA cut‐off of > 3.9 μg/mL (both upper limits of the laboratory reference intervals determined in validation studies) were used as being indicative of systemic inflammation. Serum CRP and SAA concentrations below the limit of blank (the lowest concentration that could be differentiated from zero) of the analyzer (2.2 mg/L for CRP and 0.3 μg/mL for SAA) were assigned values just below the minimal limit of detection of 2.199 mg/mL and 0.299 μg/mL, respectively, for statistical analysis.

### 2.5 Blood Film Review

A manual differential count was performed on archived blood smears (prepared from EDTA‐collected blood samples) by a single observer for patients that matched the inclusion criteria, using a standardized approach. A minimum of 100 cells was counted, and 100 cells per 10 000 leukocytes (as determined by the automated WCC) were counted in cases with a total leukocyte count exceeding 10 000 cells/μL [24]. The number of neutrophils exhibiting toxic change was graded (Table 1) from 1 to 3 as follows: (i) 1 = < 10% neutrophils, (ii) 2 = 11%–30% neutrophils, (iii) 3 = > 30%. Toxic change severity was also graded (Table 1) from 1 to 3 as follows: (i) 1 = mild basophilia, Dohle bodies; (ii) 2 = moderate basophilia, Dohle bodies, foamy cytoplasm; and (iii) 3 = marked basophilia, foamy cytoplasm, and toxic granules. The toxic change grade was calculated by adding the toxic change number (1–3) and the toxic change severity (1–3). For determination of left shift, band neutrophils were identified when the width of their nucleus was mostly uniform overall and when indentations in the nucleus were below one‐third of their maximal width [25].

**TABLE 1:** Grading neutrophil toxic change.

Score	1	2	3
Number of neutrophils with toxic change	Few = up to 10%	Moderate = 11%–30%	Many = > 30%
Severity of toxic change in neutrophils	1 = mild basophilia, Dohle bodies	2 = moderate basophilia, Dohle bodies, foamy cytoplasm	3 = Marked basophilia, foamy cytoplasm, toxic granules

*Note:* The number of neutrophils exhibiting toxic change were graded from 1 to 3 as follows: (i) 1 = < 10% neutrophils (ii) 2 = 11%–30% neutrophils (iii) 3  = > 30%. Toxic change severity of neutrophils was also graded from 1 to 3 as follows: (i) 1 = mild basophilia, Dohle bodies; (ii) 2 = moderate basophilia, Dohle bodies, and foamy cytoplasm; and (iii) 3 = marked basophilia, foamy cytoplasm, toxic granules. Toxic change grade was calculated by adding the toxic change number (1–3) and toxic change severity (1–3).

### 2.6 Assessment of Intra‐Assay Variability and the Effects of Sample Aging on Cell Population Data

For preliminary assessment of intra‐assay variability, hematology samples from dogs and cats with low, medium, and high CPD values were prospectively selected and run 5 times on the same day. Low, medium, and high values for NE‐SFL in dogs were defined as < 40.18, 40.18–43.8, and > 44.5, while in cats, low, medium, and high NE‐SFL values were defined as < 34.9, 35–39, and > 40.4. Low, medium, and high values for NE‐SSC in dogs were defined as < 99.2, 101.6–105.6, and > 106.9, and low, medium, and high NE‐SSC values in cats were defined as < 107.2, 108.1–112.1, and > 112.2. These values were determined based on the lower, median, and upper quartiles of NE‐SSC and NE‐SFL values collected as part of this study. For assessment of the effect of aging on CPD parameters, healthy dogs and cats with a normal hemogram were prospectively selected. Dogs and cats were analyzed separately. Blood samples were run at *t*0, *t* + 24 h, *t* + 48 h, and *t* + 72 h on the Sysmex XN‐V hematology analyzer, and CPD values are recorded. Samples were stored at 4°C between runs, in accordance with hematology sample storage guidelines for repeat patient testing quality control [26]. Quality control was performed daily using two levels of manufacturer‐supplied quality control material, run five times, which provided an estimation of interassay variability. Additionally, the laboratory is a participant in the External Quality Control (EQA) scheme. The coefficient of variation (CV%) for both intra‐assay variability and aging was calculated using Prism 10.

### 2.7 Statistical Analysis

Statistical analysis was performed using GraphPad Prism 10. The Kolmogorov–Smirnov test demonstrated that all data were not normally distributed; therefore, nonparametric tests were used. Comparisons between groups were carried out using a Mann–Whitney test. Where more than two groups were present, a Kruskal‐Wallis test with a Dunn multiple comparison test was used. Correlations between NE‐SFL and NE‐SCC and other hematological/biochemical parameters were assessed using Spearman's correlation coefficient. Receiver operating characteristic curve (ROC) analysis was performed, and the area under the curve (AUC) was calculated to determine the diagnostic performance of NE‐SSC and NE‐SFL for the detection of systemic inflammation, which was defined as CRP (for dogs) or SAA (for cats) concentrations above the respective cut‐off. To calculate the optimum NE‐SSC and NE‐SFL cut‐off points for detecting systemic inflammation, the Youden index was determined [27]. The likelihood ratio was calculated based on the optimal cut‐off (as determined by the Youden Index) of both NE‐SFL and NE‐SSC. Data from dogs and cats were analyzed separately in all cases. A *p*‐value of < 0.05 was considered statistically significant in all cases.

## 3 Results

### 3.1 Study Population Analysis

A total of 882 dogs and 216 cats met the inclusion criteria. Of the dogs, 339 were neutered males, 142 were intact males, 322 were neutered females, and 79 were intact females. The median age of the canine population was 7 years (range: 9 weeks–16 years). Crossbreeds were most common (*N* = 188), with other common breeds including Cocker Spaniel (*N* = 61), Labrador (*N* = 55), Springer Spaniel (*N* = 45), French Bulldog (*N* = 41), Golden Retriever (*N* = 28), Border Terrier (*N* = 26), and Jack Russell Terrier (*N* = 22).

Of the cats, 117 were neutered males, 6 were intact males, 87 were neutered females, and 5 were intact females. The median age of the feline population was 9 years (range: 3 months–20 years). The most common breed was Domestic (*N* = 152), which included both domestic short‐ and long‐haired breeds. Other breeds included British Shorthaired (*N* = 11), Maine Coon (*N* = 8), Ragdoll (*N* = 7), Siamese (*N* = 5), Crossbreed (*N* = 5), Bengal (*N* = 4), Burmese (*N* = 4), and Oriental Shorthaired (*N* = 3).

### 3.2 Neutrophil Cell Population Data Parameters in Dogs and Cats With Systemic Inflammation

**FIGURE 1:**
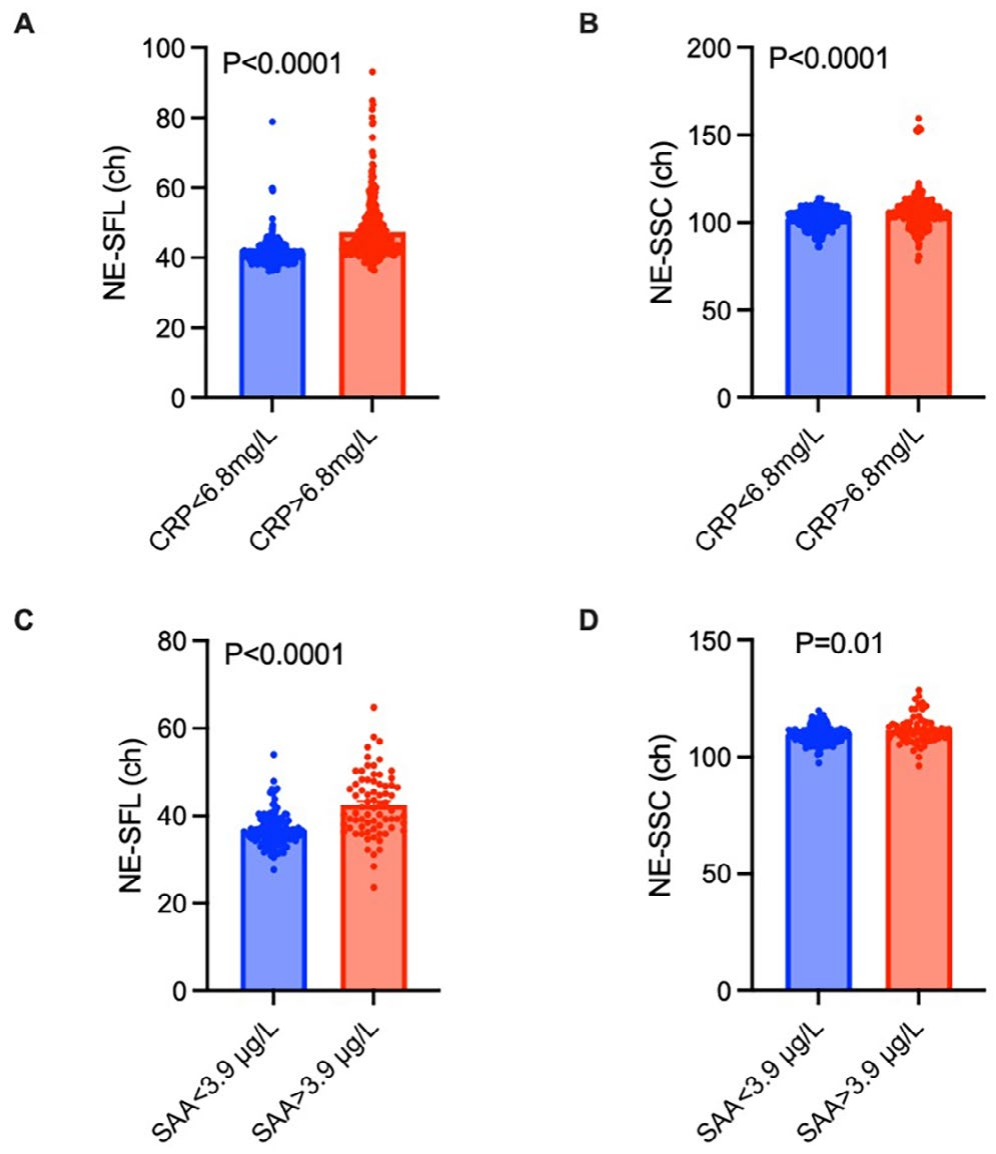
Comparison of NE‐SFL and NE‐SCC levels in dogs and cats with and without systemic inflammation. In dogs, CRP concentrations > 6.8 mg/L indicated systemic inflammation, whereas in cats, SAA concentrations > 3.9 μg/L indicated systemic inflammation. (A) NE‐SFL levels in dogs with and without systemic inflammation (*n* = 582, *n* = 300) (B) NE‐SSC in dogs with and without systemic inflammation (*n* = 582, *n* = 300) (C) NE‐SFL in cats with and without systemic inflammation (*n* = 144, *n* = 72) (D) NE‐SFL in cats with and without systemic inflammation (*n* = 144, *n* = 72). Statistical analysis was performed with a Mann–Whitney test. Error bars indicate the standard deviation. *p < 0.05.

In dogs with evidence of systemic inflammation (CRP > 6.8 mg/L), NE‐SFL (as measured by arbitrary light scattering units [ch]) was significantly elevated compared to dogs with normal serum CRP concentrations (36.1 ch [min–max, 27.7–54 ch] vs. 41.5 ch [min–max, 23.6–64.8 ch]; Figure 1A; *p* < 0.0001). Additionally, NE‐SSC (as measured by arbitrary light scattering units [ch]) was significantly elevated in dogs with systemic inflammation (109.8 ch [min–max, 97.4–119.8 ch] vs. 111.1 ch [min–max, 96.2–128.5 ch], Figure 1B; *p* < 0.0001).

**FIGURE 2:**
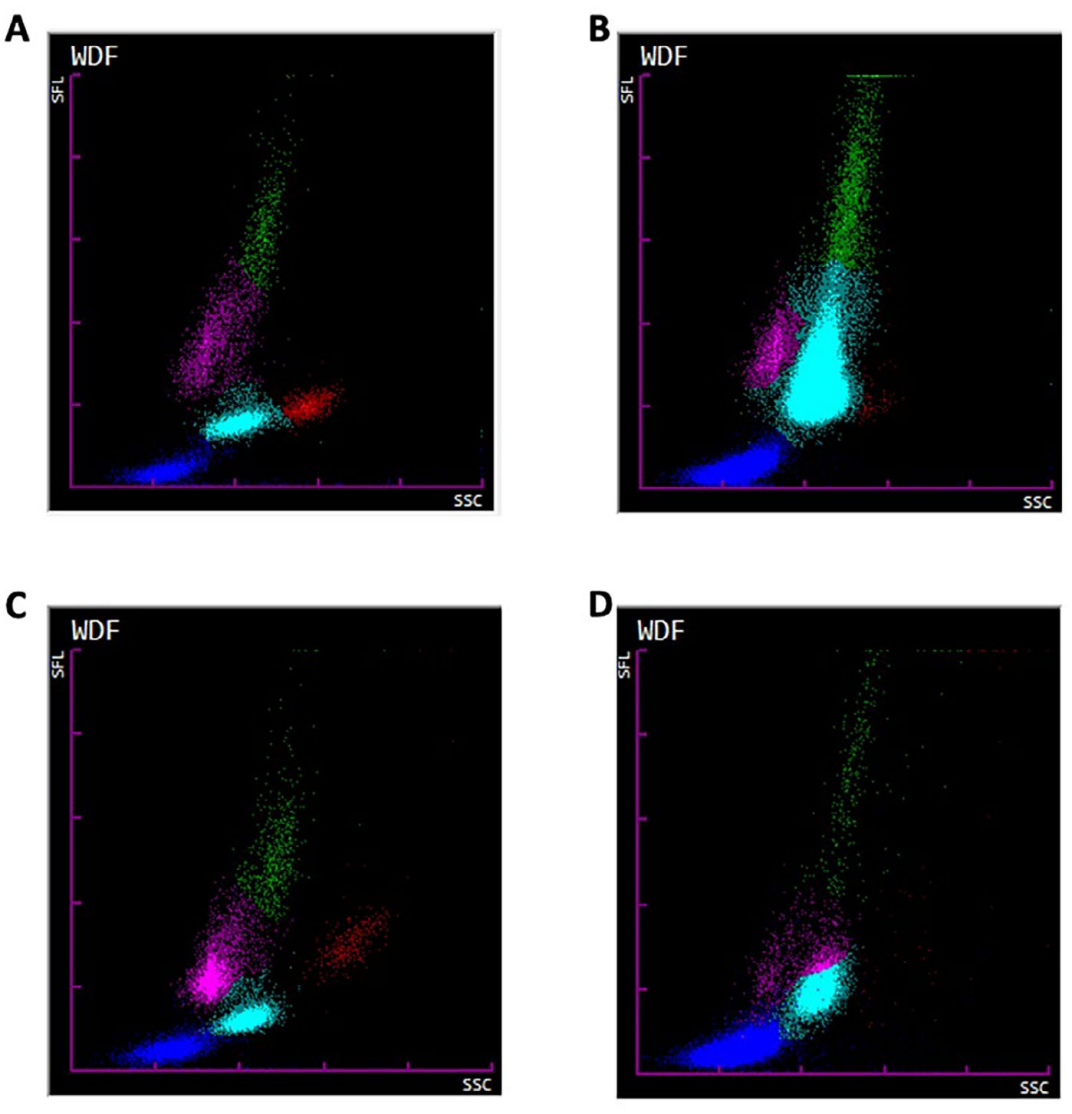
Representative Sysmex XN hematology scatterplots from dogs and cats with high and low NE‐SFL values. Scatterplots from (A) A dog with low NE‐SFL (39.5 ch) (B) A dog with high NE‐SFL (60.7 ch) (C) A cat with low NE‐SFL (31.5 ch) (D) A cat with high NE‐SFL (50.3 ch).

In cats with evidence of systemic inflammation (SAA concentrations > 3.9 μg/L), NE‐SFL was also significantly elevated compared to cats without evidence of systemic inflammation (41.5 ch [min–max, 23.6–64.8 ch] vs. 36.1 ch [min–max, 27.7–54.0 ch], Figure 1C; *p* < 0.0001). Similarly, NE‐SSC was significantly elevated in cats with systemic inflammation (111.1 ch [min–max, 96.2–128.5 ch] ch vs. 109.8 ch [min–max, 97.4–119.8 ch]; Figure 1D; *p* < 0.016). Representative Sysmex XN scatterplots from dogs and cats with high and low NE‐SFL values are shown (Figure 2).

### 3.3 Establishing Neutrophil Cell Population Data Cut‐Off Points for Detecting Systemic Inflammation

**FIGURE 3:**
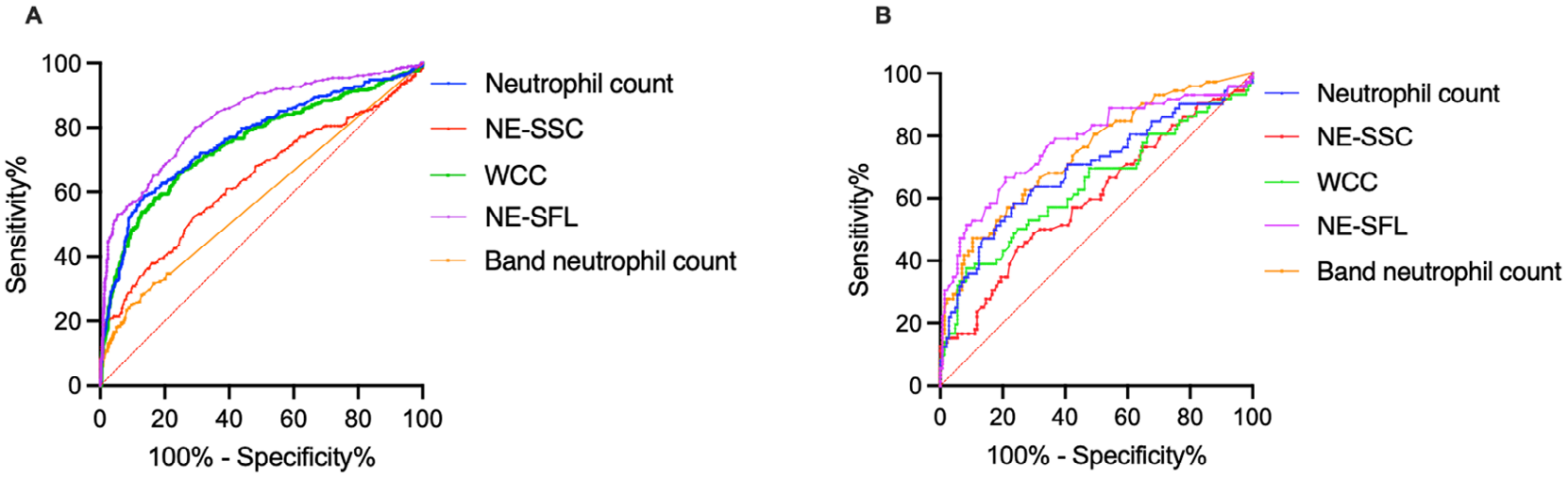
Comparison of the diagnostic sensitivity and specificity of NE‐SSC and NE‐SFL with other inflammatory markers. Receiver operating characteristic analysis (ROC) curves comparing the diagnostic sensitivity and specificity of inflammatory markers in dogs and cats (A) ROC curve comparing the sensitivity and specificity of neutrophil count, NE‐SSC, WCC, NE‐SFL, and band neutrophil count in dogs (*N* = 882) (B) ROC curve comparing the sensitivity and specificity of neutrophil count, NE‐SSC, WCC, NE‐SFL, and band neutrophil count in cats (*N* = 216). *p < 0.05.

The ROC curve AUCs for NE‐SFL, NE‐SSC, WCC, neutrophil count, and band neutrophil counts as predictors of systemic inflammation in dogs (based on serum CRP concentration) were 0.82 (95% CI, 0.79–0.85); 0.64 (95% CI, 0.6–0.68); 0.74 (95% CI, 0.71–0.78); 0.75 (95% CI, 0.72–0.79), and 0.57 (95% CI, 0.53–0.61), respectively (Table 2, Figure 3A). The optimal NE‐SFL cutoff point of 41.7 ch as a predictor of systemic inflammation in dogs corresponded to a sensitivity of 80% and a specificity of 66% (Table 2). The optimal NE‐SSC cutoff point of 104.1 ch as a predictor of systemic inflammation in dogs corresponded to a sensitivity of 61% and a specificity of 60% (Table 2).

**TABLE 2:** Cut‐off values and proposed reference intervals for NE‐SFL and NE‐SSC in dogs and cats.

CPD parameter	Species	*N*	AUC	95% confidence interval	Optimal cut‐off value (ch)	Sensitivity %	Specificity %	Likelihood ratio (Youden cut‐off)
NE‐SFL	Canine	882	0.82	0.79–0.85	> 41.7	80%	66%	2.4
NE‐SSC	Canine	882	0.64	0.6–0.68	> 104.1	61%	60%	1.5
WCC	Canine	882	0.74	0.71–0.78	> 10.33	68%	70%	2.3
Neutrophil count	Canine	882	0.75	0.72–0.79	> 7.4	68%	72%	2.4
Band neutrophil count	Canine	882	0.57	0.53–0.61	> 0.5	34%	78%	1.6
NE‐SFL	Feline	216	0.77	0.70–0.84	> 37.4	75%	67%	2.2
NE‐SSC	Feline	216	0.59	0.51–0.68	> 109.9	60%	51%	1.2
WCC	Feline	216	0.63	0.55–0.72	> 10.11	67%	54%	1.4
Neutrophil count	Feline	216	0.69	0.61–0.77	> 7.5	69%	60%	1.7
Band neutrophil count	Feline	216	0.74	0.67–0.81	> 0.42	68%	65%	1.9

*Note:* Receiver operating characteristic analysis (ROC) curves were used to calculate the area under the curve (AUC) with a 95% confidence interval. An optimal cut‐off value for NE‐SFL, NE‐SSC, WCC, neutrophil count, and band neutrophil count in dogs and cats was calculated from the ROC curve alongside the associated sensitivity and specificity. The likelihood ratio was calculated based on the Youden Index cut‐off value.

The ROC curve AUCs for NE‐SFL, NE‐SSC, WCC, neutrophil count, and band neutrophil counts as predictors of systemic inflammation in cats (based on serum SAA concentration) were 0.77 (95% CI, 0.70–0.84); 0.59 (95% CI, 0.51–0.68); 0.63 (95% CI, 0.55–0.72); 0.69 (95% CI, 0.61–0.77), and 0.74 (95% CI, 0.67–0.81), respectively (Table 2, Figure 3B). The optimal NE‐SFL cutoff point of 37.4 ch as a predictor of systemic inflammation in cats corresponded to a sensitivity of 75% and a specificity of 67% (Table 2). The optimal NE‐SSC cutoff point of 109.9 ch as a predictor of systemic inflammation in cats corresponded to a sensitivity of 60% and a specificity of 51% (Tables 2 and 3).

**TABLE 3:** Intra‐assay variability of CPD parameters in dogs and cats.

CPD parameter	Species	Low analyte level	Medium analyte level	High analyte level
		Median (minimum–maximum)	Median (minimum–maximum)	Median (minimum–maximum)
NE‐SFL (ch)	Canine	39.5 (39.3–40.6)	41.5 (41.4–43.2)	50.4 (49–51.6)
NE‐SSC (ch)	Canine	95.7 (95.4–95.8)	102.5 (101.4–102.8)	112.3 (112.2–112.6)
NE‐SFL (ch)	Feline	34.4 (33.9–34.8)	38.7 (37.2–39)	41.6 (40.1–41.7)
NE‐SSC (ch)	Feline	108 (107.4–108.3)	108.1 (107–108.6)	113.9 (113.8–114.3)
Control NE‐SFL (ch)	—	94.1 (93.8–96.2)	—	90.6 (89.6–90.9)
Control NE‐SSC (ch)		170.4 (170–170.5)		170.7 (170.2–171.1)

*Note:* Representative descriptive statistics data of intra‐assay variability of samples with low, medium, and high CPD parameters, run 5 times on the same day. Values represent the median and range (minimum to maximum). Controls represent quality control material provided by the manufacturer.

### 3.4 Correlation of NE‐SFL and NE‐SCC With Hematological and Acute Phase Protein Markers of Inflammation

In dogs, NE‐SFL had a moderate positive correlation with serum CRP concentration (*r*_*s*_ = 0.56; *p* = < 0.0001), and a weak [28] positive correlation with WCC (*r*_*s*_ = 0.35; *p* = < 0.0001), neutrophil count (*r*_*s*_ = 0.36; *p* = < 0.0001), band neutrophil number (*r*_*s*_ = 0.21; *p* = < 0.0001), and toxic change grade (*r*_*s*_ = 0.14; *p* = < 0.0001). Similarly, in dogs, NE‐SCC showed a weak positive correlation with serum CRP concentration (*r*_s_ = 0.25; *p* = < 0.0001), WCC (*r*_*s*_ = 0.12; *p* = 0.0005), neutrophil count (*r*_*s*_ = 0.11, *p* = 0.0007), and band neutrophil count (*r*_*s*_ = 0.09, *p* = 0.005). However, NE‐SCC was not significantly correlated with toxic change grade.

In cats, NE‐SFL showed a moderate positive correlation with SAA concentration (*r*_*s*_ = 0.5; *p* = < 0.0001) and a weak positive correlation with WCC (*r*_*s*_ = 0.15; *p* = 0.02), neutrophil count (*r*_*s*_ = 0.15; *p* = 0.02), and band neutrophil count (*r*_*s*_ = 0.27; *p* = < 0.0001). However, NE‐SFL was not significantly correlated with toxic change grade. Similarly, in cats, NE‐SCC showed a weak positive correlation with SAA (*r*_*s*_ = 0.16; *p* = 0.013). However, there was no significant correlation between NE‐SCC and WCC, neutrophil count, band neutrophil number, and toxic change grade.

### 3.5 Intra‐Assay, Interassay Variability and the Effects of Sample Aging on Cell Population Data Parameters

Data regarding intra‐assay variability (low, medium, and high values) in canine (*N* = 3), feline (*N* = 3), and control (*N* = 2) samples are shown in the supplemental data. In dogs, the overall intra‐assay variability of NE‐SFL and NE‐SSC was 1.4%–2% and 0.1%–0.5%, respectively. The median [range] low, medium, and high analyte levels for NE‐SFL were 39.5 ch [39.3–40.6 ch], 41.5 ch [41.4–43.2 ch], and 50.4 ch [49–51.6 ch], respectively. While the median [range] low, medium, and high analyte levels for NE‐SSC were 95.7 ch [95.4–95.8 ch], 102.5 ch [101.4–102.8 ch], and 112.3 ch [112.2–112.6 ch], respectively. In cats, overall the intra‐assay variability of NE‐SFL and NE‐SSC was 0.9%–2% and 0.17%–0.5%, respectively. The median [range] low, medium, and high analyte levels for NE‐SFL were 34.4 ch [33.9–34.8 ch], 38.7 ch [37.2–39 ch], and 41.6 ch [40.1–41.7 ch], respectively. While the median [range] low, medium, and high analyte levels for NE‐SSC were 108 ch [107.4–108.3 ch], 108.1 ch [107–108.6 ch], and 113.9 ch [113.8–114.3 ch], respectively.

The effect of sample aging on CPD values in canine (*N* = 5) and feline (*N* = 3) blood run at *t*0, *t* + 24 h, *t* + 48 h, and *t* + 72 h was determined (Supplemental data). The CV% for CPD parameters over time was determined: canine NE‐SFL (2%–6.8%), canine NE‐SSC (1.5%–3%), feline NE‐SFL (1.8%–10.4%), feline NE‐SSC (2.2%–3.1%).

The interassay variability, assessed from two levels of manufacturer‐supplied Quality Control Material (*N* = 2) was 0.84%–1% for NE‐SFL and 0.16%–0.22% for NE‐SSC (Supplemental data). The median [range] low and high analyte levels for NE‐SFL were 94.1 ch [93.8–96.2 ch] and 90.6 ch [89.6–90.9 ch], respectively, while the median [range] low and high analyte levels for NE‐SSC were 170.4 ch [170–170.5 ch] and 170.7 ch [170.2–171.1 ch], respectively.

## 4 Discussion

This paper explores, for the first time, the ability of CPD parameters NE‐SSC and NE‐SFL to detect systemic inflammation in dogs and cats. Here we show that both NE‐SSC and NE‐SFL values are significantly increased in dogs and cats with systemic inflammation and positively correlated with APP concentrations and established hematological parameters.

The literature has shown that neutrophil CPD parameters are useful in detecting systemic inflammation secondary to sepsis in humans [18, 29]. Some studies have explored the diagnostic utility of CPD in other scenarios. For example, in a study by Nguyen et al., NE‐SFL was increased in patients undergoing cardiac surgery with cardiopulmonary bypass, suggesting that these CPD values may also reflect the activation of leukocytes in sterile inflammation [30]. However, as of yet, the diagnostic utility of CPD analytes for detecting systemic inflammation across a range of different diseases has not been examined in humans and animals. In the present study, both NE‐SSC and NE‐SFL were significantly increased in dogs and cats with systemic inflammation of many causes. Since increases in NE‐SFL suggest higher neutrophil RNA/DNA content (and there is some, albeit limited, evidence to suggest that NE‐SSC also indicates left shift/toxic change [19]), these parameters might be useful in the laboratory setting, and when abnormally increased, might be suggestive of blood film abnormalities prior to the microscopic examination of a blood smear. As automated indices, they are more objective than manual blood film evaluation and may be of benefit when the latter is delayed or when trained observers are unavailable.

ROC analysis revealed that NE‐SFL in dogs was superior to NE‐SSC, WCC, and band neutrophil count in detecting systemic inflammation (based on the lack of overlap in AUC 95% confidence intervals), while in cats it was only superior to NE‐SSC. In both species, the use of the optimal cut‐offs determined via the Youden Index was associated with good sensitivity but lower specificity for the detection of systemic inflammation. As a screening test, where sensitivity should be prioritized, the established cut‐offs for NE‐SFL are potentially clinically useful in both dogs and cats and could prompt the measurement of APPs. The sensitivity and specificity of NE‐SSC were inferior to NE‐SFL in dogs and cats and failed to demonstrate superiority over established inflammatory markers (with the exception of band neutrophil count in dogs), thereby limiting its potential as a diagnostic test.

In both dogs and cats, CPD parameters were determined to have low and acceptable intra‐assay variability. Furthermore, assessment of CPD stability over time suggests that CPD parameters remain acceptably stable over 72 h and may therefore be of use in commercial laboratories affected by delays in sample processing with shipment [31]. Additionally, sample storage is known to result in false classification of neutrophil toxic change when smear preparation is delayed [32], and therefore, CPD parameters may facilitate a more accurate assessment of inflammatory status in these cases. Notably, however, future studies should aim to assess neutrophil granularity over time in blood samples in which toxic changes of various intensities are noted in order to determine if both toxicity and granularity increase proportionally over time. Our findings indicate that NE‐SFL, in particular, has some value as an inflammatory marker [10, 32]. One key advantage of CPD parameters is that they do not require additional sampling and are available at no extra cost when hematological analysis is performed on a Sysmex analyzer. However, other inflammatory markers, such as APPs, are increasingly more readily available than Sysmex analyzers (which are only available in large institutions and laboratories), limiting the utility of CPD parameters in clinical practice, especially when compared to WCC, band neutrophil count, and blood smear evaluation of neutrophil toxicity. An important future objective could be to assess similar parameters available within the service menu of newer in‐clinic analyzers, for example, the ProCyte Dx (IDEXX Laboratories). Although it was beyond the scope of this retrospective study, it will be an important and interesting additional future objective to assess the capacity of CPD parameters, particularly NE‐SFL in addition to other CPD values investigated in humans, such as NE‐WY [20], to offer prognostic information and to guide therapeutic efforts in specific diseases and to look at any potential effects of age, sex, and breed on these CPD parameters. Additionally, prospective determination of reference intervals for CPD parameters will be useful in further investigating their clinical utility.

This study had several limitations. First, this was a single‐center retrospective study in which patient numbers could not be controlled, resulting in approximately three times as many dogs as cats being included (a reflection of the patient population at the hospital). Furthermore, it is possible that confounding factors, including multiple comorbidities and treatment effects such as the administration of anti‐inflammatory medications, which were beyond the scope of this study to evaluate, could have affected the results. Additionally, the stability of CPD parameters over time was only assessed in healthy animals and not in dogs and cats with an abnormal hemogram, and assessing stability in this population will be an important future objective. The sample size for the stability over time study was also low in cats due to insufficient sample volume to facilitate repeat testing over time. For the purposes of the study, APP concentrations above the reference intervals were used as the “gold standard” for the diagnosis of systemic inflammation; however, as with all diagnostic tests, it remains possible (although unlikely, based on published sensitivity and specificity of APPs [10, 33]) that some individuals may have been misclassified using this approach alone. While 100 cells were always counted when performing white cell differential counts on blood smears, the greatest discrepancy between automated and manual differential counts occurred in patients with leukopenia. It is therefore possible that this approach may have introduced sampling errors in these patients, as fewer cells were available for evaluation. Another important consideration is that CPD values are measured on cells that the analyzer software classifies as neutrophils. However, hematology analyzers such as Sysmex are known to misclassify neutrophils as other inflammatory cells, such as monocytes or lymphocytes, particularly during inflammatory states [31, 34]. Therefore, the observed diagnostic limitations of these CPD parameters compared to APPs could in part be explained by misclassification errors of the Sysmex hematology analyzer. Preliminary assessment of the cases in this study in which the discrepancy between automated and manual neutrophil counts was greatest found that manual regating had minimal impact on CPD parameters; however, further prospective studies could more thoroughly investigate the effect of manual regating on CPD parameters.

## 5 Conclusion

In conclusion, this study suggests that CPD parameters, particularly NE‐SFL, show promise as markers of systemic inflammation in dogs and cats. These values could aid in the identification of samples that are likely to have blood film abnormalities and prompt APPs measurement.

## Conflicts of Interest

The authors declare no conflicts of interest.

## Supporting information


Data S1.
