# Supplementary material for: The Value of Neutrophil Cell Population Data Parameters as Markers of Systemic Inflammation in Dogs and Cats
Source: Vet Clin Pathol. 2025 Jun 6;54(2):78–86. doi: 10.1111/vcp.70029 (PMC12289122; doi:10.1111/vcp.70029)
Supplement: Supplementary file 1 — Data S1. [file VCP-54-78-s001.docx]

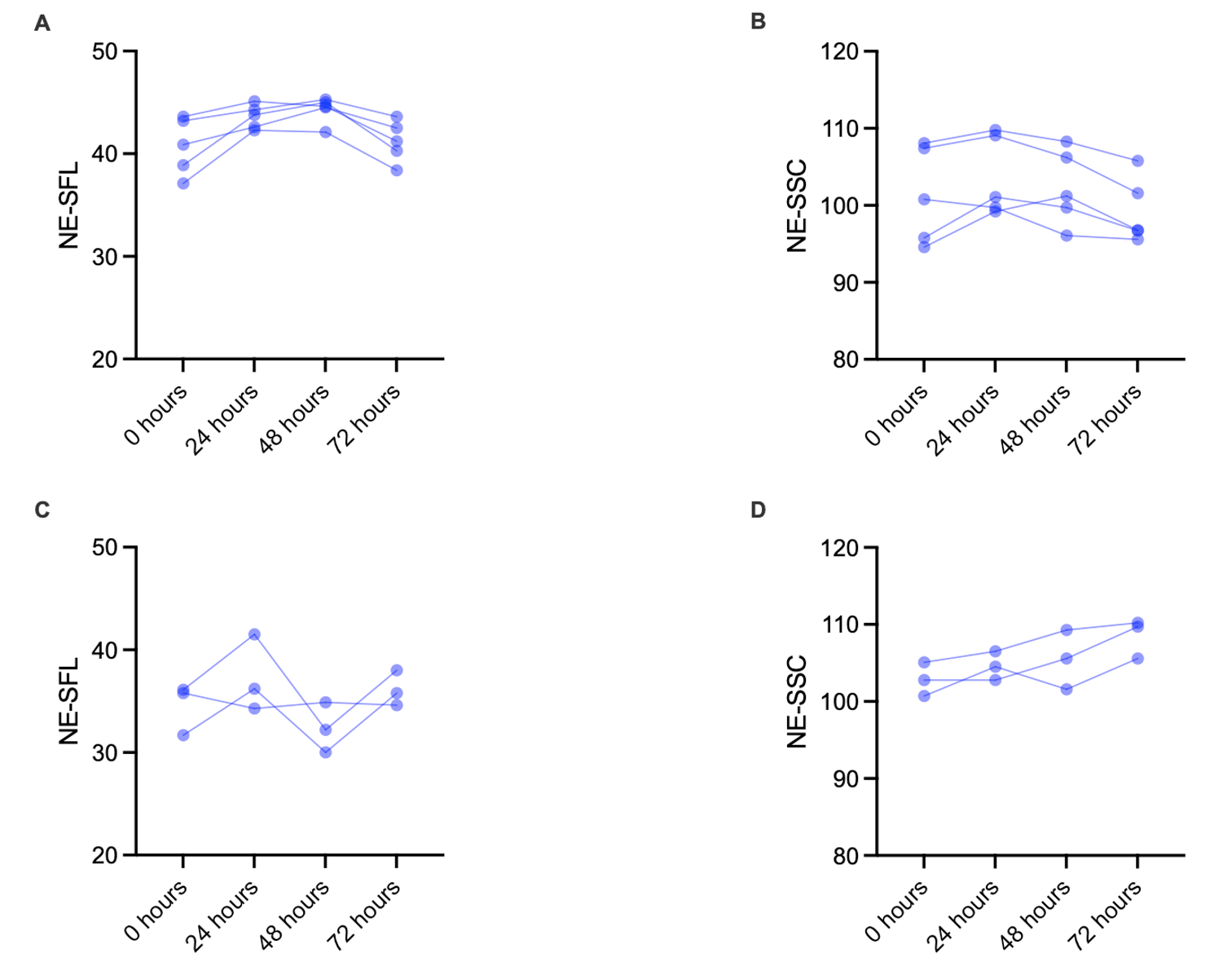


**The effects of sample aging on cell population data parameters**

Samples from dogs (N=5) and cats (N=3), stored at 4°C, were run at t0, t+24hrs, t+48 hours and t+72 hours on the Sysmex XN-V hematology analyzer. CPD values over time for **(A)** Canine NE-SFL, **(B)** Canine NE-SSC, **(C)** Feline NE-SFL and **(D)** Feline NE-SSC are shown. The Coefficient of variation (CV%) for each CPD parameter was determined.


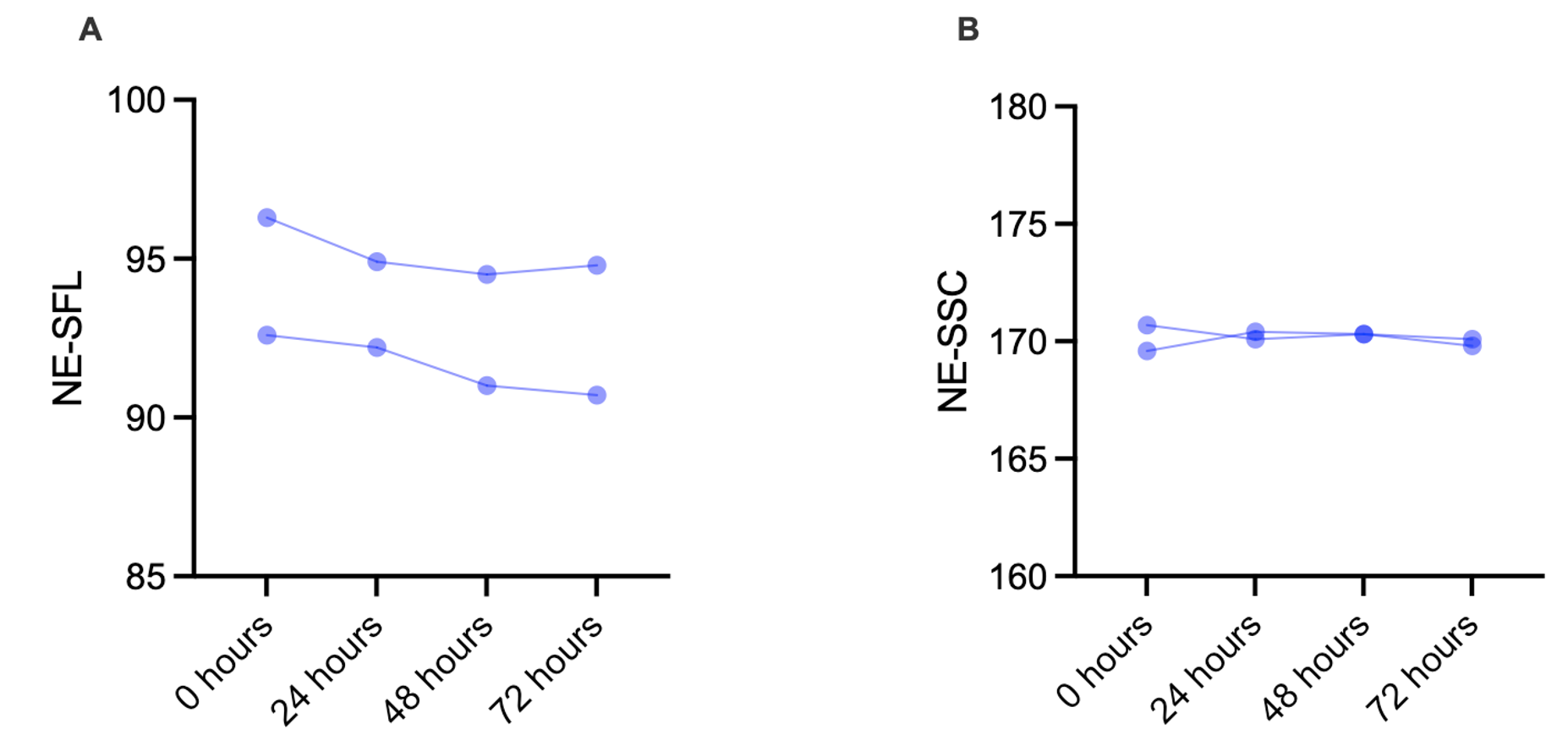


**Inter-assay variability of cell population data parameters**

Two levels of manufacturer supplied Quality Control Material (N=2), stored at 4°C, were run at t0, t+24hrs, t+48 hours and t+72 hours. Inter-assay variability for **(A)** NE-SFL and **(B)** NE-SSC are shown. The inter-assay variability for NE-SFL was 0.84-1% and the inter-assay variability of NE-SSC was 0.16-0.22%.
